# Antiproliferative Scalarane-Based Metabolites from the Red Sea Sponge *Hyrtios erectus*

**DOI:** 10.3390/md14070130

**Published:** 2016-07-08

**Authors:** Sameh S. Elhady, Ahmed M. Al-Abd, Ali M. El-Halawany, Abdulrahman M. Alahdal, Hashim A. Hassanean, Safwat A. Ahmed

**Affiliations:** 1 Department of Pharmacognosy, Faculty of Pharmacy, Suez Canal University, Ismailia 41522, Egypt; ssahmed@kau.edu.sa (S.S.E.); hashem_omar@pharm.suez.edu.eg (H.A.H.); 2 Department of Natural Products and Alternative Medicine, Faculty of Pharmacy, King Abdulaziz University, Jeddah 21589, Saudi Arabia; ahalawany2003@yahoo.com; 3 Pharmacology Department, Medical Division, National Research Centre, Giza 12622, Egypt; ahmedmalabd@pharma.asu.edu.eg; 4 Department of Pharmacology and Toxicology, Faculty of Pharmacy, King Abdulaziz University, Jeddah 21589, Saudi Arabia; 5 Pharmacognosy Department, Faculty of Pharmacy, Cairo University, Kasr el-Aini street, Cairo 11562, Egypt; 6 Department of Clinical Pharmacy, Faculty of Pharmacy, King Abdulaziz University, Jeddah 21589, Saudi Arabia; aalahdal2@hotmail.com

**Keywords:** Red Sea sponge, *Hyrtios erectus*, scalarane framework, cell based assay, antiproliferative activity

## Abstract

Two new sesterterpenes analogs, namely, 12-acetoxy,16-*epi*-hyrtiolide (**1**) and 12β-acetoxy,16β-methoxy,20α-hydroxy-17-scalaren-19,20-olide (**2**), containing a scalarane-based framework along with seven previously reported scalarane-type sesterterpenes (**3**–**9**) have been isolated from the sponge *Hyrtios erectus* (order Dictyoceratida) collected from the Red Sea, Egypt. The structures of the isolated compounds were elucidated on the basis of their spectroscopic data and comparison with reported NMR data. Compounds **1**–**9** exhibited considerable antiproliferative activity against breast adenocarcinoma (MCF-7), colorectal carcinoma (HCT-116) and hepatocellular carcinoma cells (HepG2). Compounds **3**, **5** and **9** were selected for subsequent investigations regarding their mechanism of cell death induction (differential apoptosis/necrosis assessment) and their influence on cell cycle distribution.

## 1. Introduction

**Figure 1:**
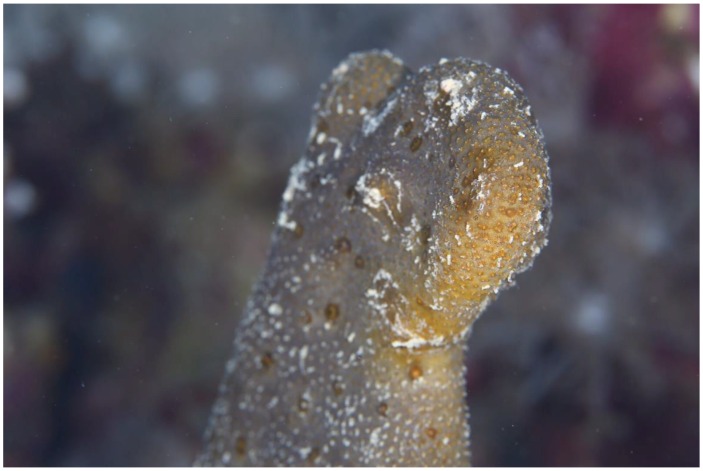
Red Sea sponge *Hyrtios erectus* (Underwater photograph).

Marine organisms are well known as a rich source of novel and structurally diverse natural products with useful biological activities [1,2]. Scalarane sesterterpenes have been identified from sponges and nudibranchs [3]. As common metabolites, scalarane sesterterpenes have been isolated from marine sponges belonging to the order Dictyoceratida [1]. Research regarding the physiological activity of these scalarane-type sesterterpenes is of particular interest. Scalarane-type sesterterpenes display a variety of pharmacological activities such as cytotoxic [4,5,6,7,8,9,10,11], antitubercular [12], antimicrobial [13,14], anti-inflammatory [15,16], antifeedant [17,18], ichthyotoxic [19], platelet aggregation inhibition [20,21], protease inhibition and nerve growth factor synthesis-stimulation [3]. The marine sponges *Hyrtios erectus* (order Dictyoceratida, family Thorectidae) [22] have been proven to be a rich source of secondary metabolites, including sesterterpenes [4,5,12,23,24], sesquiterpenes [25,26,27], macrolides [28,29], indole and β-carboline alkaloids [30,31,32,33,34]. In the course of our ongoing research program on bioactive secondary metabolites from Red Sea marine invertebrates, we have investigated the bioactive extract of the Red Sea sponge *Hyrtios erectus* (Figure 1). Recently, chemical investigation of the lipophilic fraction of the same sponge afforded a new pentacyclic nitrogen containing scalarane, named 24-methoxypetrosaspongia C [35].

Antiproliferative bioassay guided fractionation of the extract allowed the identification of sesterterpenes possessing a scalarane-type framework including two new compounds (**1**) and (**2**) together with the known compounds 12β,20-dihydroxy-16β-acetoxy-17-scalaren-19,20-olide (**3**) [36], Sesterstatin 7 (**4**) [12], Heteronemin (**5**) [37], Scalarolide (**6**) [17], 12-*epi*-24-deoxyscalarin (**7**) [38], Scalarolide acetate (**8**) [14] and 12-deacetyl-12,18-di-*epi*-scalaradial (**9**) [17]. The antiproliferative activity of compounds **1**–**9** against breast adenocarcinoma (MCF-7), colorectal carcinoma (HCT-116) and hepatocellular carcinoma cells (HepG2) was evaluated.

## 2. Results and Discussion

### 2.1. Purification of Compounds **1**–**9**

**Figure 2:**
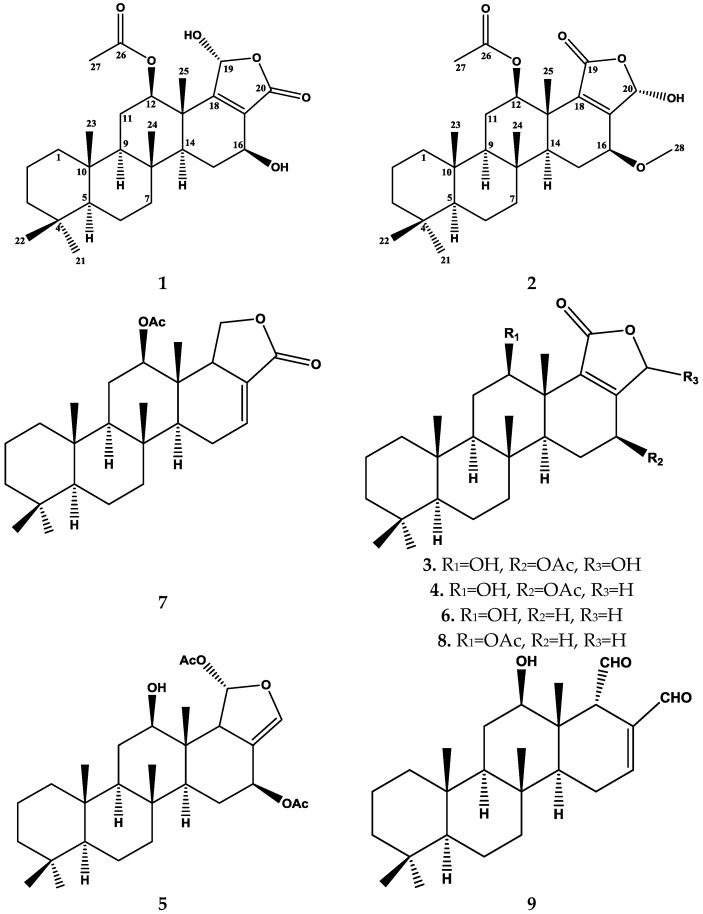
Structure of isolated compounds **1**–**9**.

Repeated chromatographic fractionation using silica gel column chromatography and final purification on C18 HPLC column of the lipophilic fraction obtained from a MeOH extract of the sponge afforded nine pure compounds (**1**–**9**), of which (**1**) and (**2**) were determined as new sesterterpenoid analogs. Compounds (**3**–**9**) were identical to scalarane type sesterterpenoids, namely, 12β,20-dihydroxy-16β-acetoxy-17-scalaren-19,20-olide (**3**) [36], Sesterstatin 7 (**4**) [12], Heteronemin (**5**) [37], Scalarolide (**6**) [17], 12-*epi*-24-deoxyscalarin (**7**) [38], Scalarolide acetate (**8**) [14] and 12-deacetyl-12,18-di-*epi*-scalaradial (**9**) [17], respectively. All of the known compounds (**3**–**9**) (Figure 2) were readily identified by extensive study of their spectral data, including ESIMS, 1D and 2D NMR data, as well as by comparison with those reported in the literature.

### 2.2. Structure Elucidation of Compounds **1**–**9**

**Figure 3:**
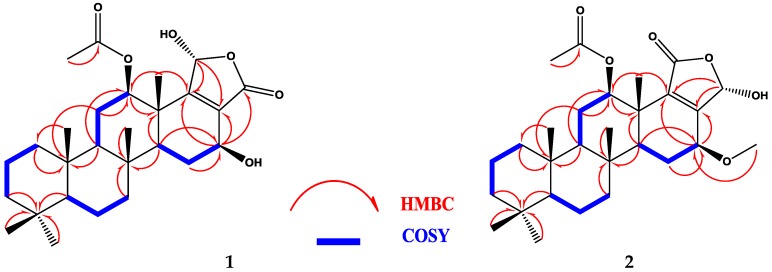
Selected COSY (correlation spectroscopy) and HMBC correlations of compounds **1** and **2**.

Compound **1** (Figure 2) was isolated and purified as amorphous solid. The HRESIMS (high-resolution electrospray ionization mass spectrometry) gave the molecular formula C_27_H_40_O_6_, which established from the positive pseudomolecular ion peak at *m/z* 461.2901 [M + H]^+^. The ^1^H NMR spectrum of compound **1** (Table 1) exhibited six methyl groups as singlets at [δ_H_ 0.80 (3H), 0.84 (6H), 0.89 (3H), 1.23 (3H), and 2.09 (3H)]. Additionally, the ^1^H NMR spectrum revealed three protons in the vicinity of the oxygen-bearing substituents δ_H_ 6.17 (s), 5.67 (dd, *J =* 9.6, 7.2 Hz) and 3.82 (dd, *J =* 16.8, 6.6 Hz) (Supplementary Materials, Figure S1). The ^13^C NMR spectrum (Table 1) exhibited signals for 27 carbons including six methyls, seven methylenes, six methines and eight quaternary carbons (Supplementary Materials, Figure S2). The ^1^H-^1^H-COSY (correlation spectroscopy) (Figure 3) and the HSQC (heteronuclear single-quantum correlation spectroscopy) NMR data analysis indicate the following partial fragments: C-1 to C-3; C-5 to C-7; C-9 to C-12; and C-14 to C-16. In addition, the correlations of H-12 (δ_H_ 3.82) with the acetyl carbon at δ_C_ 169.8 and H-16 with neighboring carbons in the HMBC (heteronuclear multiple-bond correlation spectroscopy) (Supplementary Materials, Figures S3–S5) allowed identification of a 12-acetoxy-16-hydroxyscalarane framework (Figure 3). The ^1^H and ^13^C spectral data were compatible to a large degree with those of the known scalarane sesterterpenoid hyrtiolide, [24] with the exception of an additional acetyl group δ_H_ 2.09 (3H, s); δ_C_ 21.02 (CH_3_), 169.8 (qC) present in compound **1**. The C-17/C-18 double bond was inferred by long range correlations between H_3_-25 at δ_H_ 1.23 and the quaternary olefinic carbon at δ_C_ 168.7 (C-18) and between H-16 at δ_H_ 5.67 and the olefinic carbon at δ_C_ 126.1 (C-17). Furthermore, the ^13^C chemical shifts of C-17 and C-18 indicated the location of the carbonyl at C-20 [23,24].

**Figure 4:**
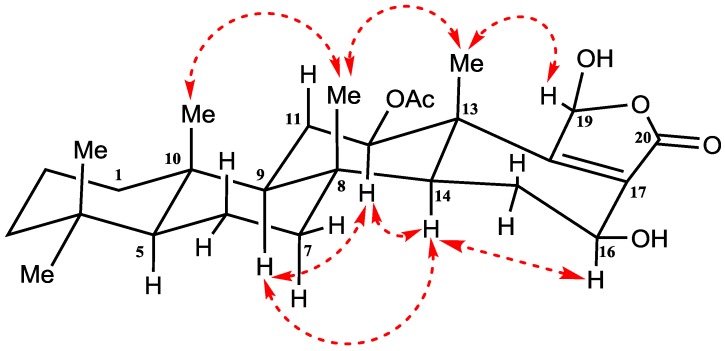
Important NOESY NMR correlations of compound **1**.

The relative configuration of H-12, H-16 and H-19 was detected by their coupling constants and confirmed by interpreting the NOESY spectrum (nuclear overhauser effect spectroscopy) (Supplementary Materials, Figure S6). The α-configuration of H-12 was deduced on the basis of the diaxial coupling of H-12 (δ_H_ 3.82; dd, *J =* 16.8 and 6.6 Hz) with H-11 and cross-peaks with α oriented H-9 and H-14 in NOESY (Figure 4). Similarly, the diaxial coupling of H-16 (δ_H_ 5.67; dd, *J =* 9.6 and 7.2 Hz) with H-15 indicates its α-configuration which was confirmed by cross-peaks with α oriented H-14 in NOESY (Figure 4). Finally, the β-configuration of H-19 was indicated by NOESY cross-peak between H-19 (δ_H_ 6.17) and Me-25 (δ_H_ 1.23). Thus, compound **1** was identified as 12-acetoxy,16-*epi*-hyrtiolide (Figure 4).

Compound **2** (Figure 2) was purified as amorphous solid. The molecular formula C_28_H_42_O_6_ was deduced from HRESIMS as well as from ^13^C NMR data. The ^1^H NMR spectrum of compound **2** (Table 2) included seven singlets, two of them at δ_H_ 2.13 (3H) and 3.47 (3H) were assigned as an acetoxy and methoxy groups, respectively. While the other remaining singlets at δ_H_ 0.80 (3H), 0.82 (3H), 0.84 (3H), 0.92 (3H) and 1.26 (3H) were belonging to the five methyl groups of a scalarane framework sesterterpene.

Additionally, the specrum displayed resonances for 21 protons including, seven methylenes, six aliphatic methines and exchangeable broad signal at δ_H_ 4.25 for OH moiety (Supplementary Materials, Figure S7). The ^13^C NMR spectrum (Table 2) displayed signals at δ_C_ 167.8 (C-19), 159.4 (C-17), 138.3 (C-18) and 94.2 (CH-20), which is characteristic for furan-derived, α,β-unsaturated-γ-hydroxybutyrolactone that is frequently present at scalarane sesterterpenes terminus [39]. In addition, the remaining signals in ^13^C NMR spectrum represent signals for 24 carbons including seven methyls, seven methylenes, five methines, and five quaternary carbons (Supplementary Materials, Figure S8). In HMBC data (Figure 3), the long-range correlations between the H-16 (δ_H_ 4.08) and the carbon atoms (δ_C_ 159.4, C-17); (δ_C_ 138.3, C-18) and (δ_C_ 57.5, C-28) determined the position of methoxy group at C-16 (Supplementary Materials, Figures S9 and S10). The location of C-17 and C-18 was confirmed by HMBC correlation between H-12 and C-18. Furthermore, the ^13^C chemical shifts of C-17 and C-18 confirmed the location of the carbonyl group at C-19 [39]. Therefore, the structure of compound **2** was elucidated as a 12-acetoxy-16-methoxyscalarane framework (Figure 3) based on the correlations of H-12 and H-16 with neighboring protons and carbons in the COSY (Supplementary Materials, Figure S11) and HMBC.

**Figure 5:**
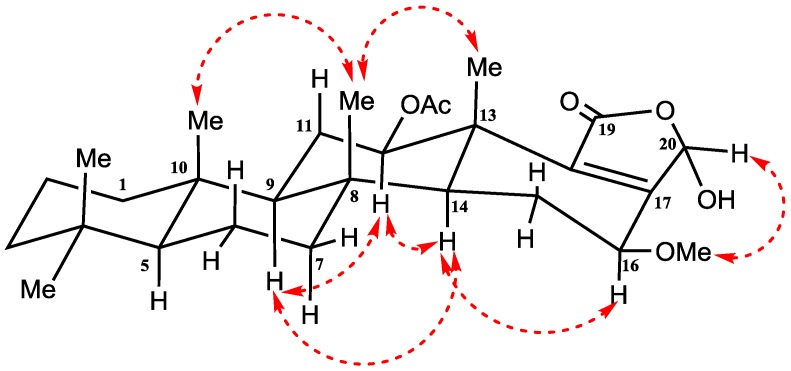
Important NOESY NMR correlations of compound **2**.

Relative configuration at C-12, C-16 and C-20 was detected and confirmed based on their coupling constants and NOESY correlations (Supplementary Materials, Figure S12). On the basis of the coupling constants, the diaxial coupling of H-12 (δ_H_ 4.88; dd, *J =* 10.8 and 3.6 Hz) with H-11 and NOESY cross-peaks with α oriented H-9 and H-14 indicate its α-configuration (Figure 5).

Similarly, the diaxial coupling of H-16 (δ_H_ 4.08; dd, *J =* 9.0 and 6.6 Hz) with H-15 indicates the α-configuration of H-16 which was confirmed by NOESY cross-peaks with the α oriented H-14 (Figure 5). Finally, cross-peaks between H-20 and β-OMe in NOESY indicate its β-configuration (Figure 5).

### 2.3. Biological Activities of the Isolated Compounds

#### 2.3.1. Antiproliferative Assessment of Compounds **1**–**9**

SRB-U assay was used to assess the antiproliferative effects of compounds **1**–**9** (Table 3) against three different tumor cell lines over concentration range 0.01–100 μM. All tested compounds showed considerable antiproliferative activity against all cell lines under investigation (MCF-7, HCT-116 and HepG2). However, HepG2 cells was relatively more sensitive while MCF-7 cells was resistant to these compounds with average IC_50_’s of 29.51 μM, 27.05 μM and 25.41 μM for MCF-7, HCT-116 and HepG2 cells, respectively.

In MCF-7 breast cancer cells, compound **5** and compound **9** showed the most potent cytotoxic profile with IC_50_s of 1.1 μM, and 3.3 μM, respectively. Compound **6** was the weakest against MCF-7 cells with IC_50_s higher than 100 μM. Other compounds (**1**, **2**, **3**, **4**, **7** and **8**) showed considerable cytotoxic profile with IC_50_s ranging from 12.7 μM to 40.3 μM (Table 3).

With respect to HCT-116 colorectal cancer cells, Compounds **3**, **5** and **9** possessed the best cytotoxic profile against HCT-116 cell line with IC_50_s of 3.5 μM, 0.7 μM and 3.4 μM, respectively. Other compounds (**1**, **2**, **4**, **7** and **8**) showed moderate cytotoxicity with IC_50_s ranging from 14.4 μM to 57.5 μM. Only compound **6** did not show any considerable cytotoxicity with IC_50_ higher than 100 μM (Table 3).

In HepG2 liver cancer cells, compounds **3**, **5** and **9** showed relatively potent cytotoxic effect with IC_50_s of 9.6 μM, 1.1 μM and 1.7 μM, respectively. Compound **6** possessed weak cytotoxicity against HepG2 cells with IC_50_s higher than 100 μM. Other compounds (**1**, **2**, **4**, **7** and **8**) showed moderate cytotoxicity with IC_50_s ranging from 15.5 μM to 42.5 μM. Accordingly, compounds **3**, **5** and **9** were selected for subsequent investigations regarding their mechanism of cell death induction (differential apoptosis/necrosis assessment) and their influence on cell cycle distribution.

#### 2.3.2. Programmed Cell Death Induced by Compounds **3**, **5** and **9** against HCT-116 Cells

**Figure 6:**
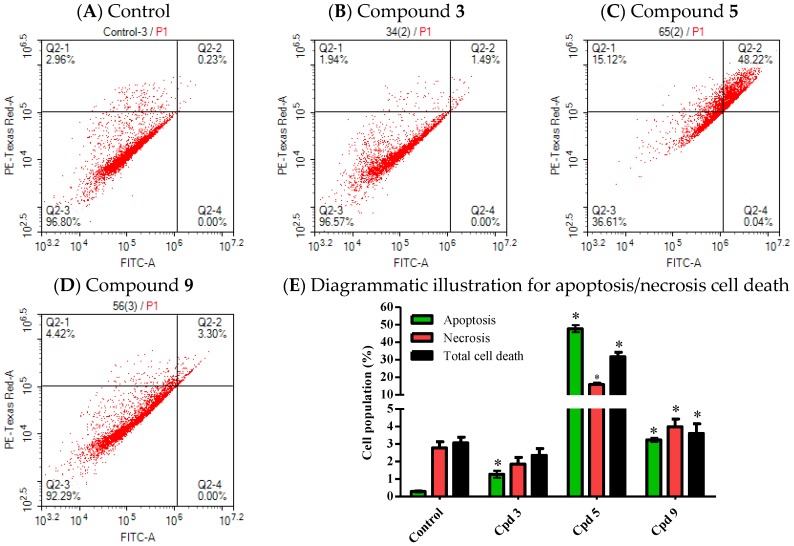
Apoptosis/necrosis analysis was assessed using annexin-V FITC/PI differential staining. HCT-116 cells were exposed to 5 µM of compounds **3** (**B**), **5** (**C**) and **9** (**D**) for 24 h and compared to control cells (**A**). Cells positive FITC, PI or FITC/PI were determined using quadrant analysis and plotted as percent of total population (**E**). Data are expressed as Mean ± SEM, *n* = 3; * significantly different from corresponding control at *p* < 0.05.

Annexin V-FITC/PI staining coupled with flowcytometry was used to assess proportion of cells undergoing necrosis or cells undergoing programmed cell death (apoptosis). HCT-116 was treated with 5 µM of compounds **3**, **5** and **9** for only 24 h and apoptosis/necrosis cell death was assessed. Compound **5** significantly increased total cell death by 20 fold compared to control. Cell death induced by compound **5** is attributed mainly to apoptosis induction; and a much lesser extent is attributed to necrosis (Figure 6A,C,E). Compound **9** significantly induced total cell death by 1.2 fold compared to control cells. Similarly, compound **9** mainly induced cell killing effect via the activation of programmed cell death rather than non-specific necrosis cell death (Figure 6A,D,E). Compound **3** was the weakest among the other selected compounds; it only induced more apoptosis with reciprocal less necrosis compared to control cells. However, total cell death induced by compound **3** was not significant from control untreated cells (Figure 6A,B,E).

After exposure of HCT-116 cells to the pre-determined IC_50_’s of selected compounds, trypan blue exclusion assay was used to confirm percent of cells with lost membrane integrity (necrosis and late apoptosis populations). After cell exposure for 72 h, compound **3** and compound **9** induced moderate membrane integrity damage indicated by 5.1% ± 1.3% and 9.5% ± 2.1% positive trypan blue cells, respectively. On the other hand, compound **5** induced profound membrane integrity damage with 67.6% ± 4.2% trypan blue positive cells (Supplementary Table S1).

Figure 6 Effect of potentially active compounds on cell death profile against HCT-116 cells.

#### 2.3.3. Influence of Compounds **3**, **5** and **9** on Cell Cycle Distribution of HCT-116 Cells

**Figure 7:**
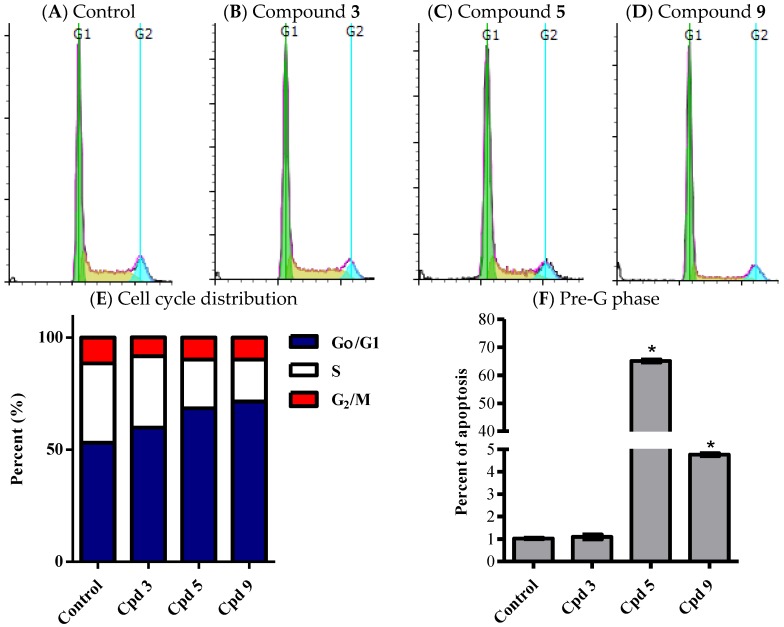
Effect of Compounds **3**, **5** and **9** on the cell cycle distribution of HCT-116 cells. The cells were exposed to Compounds **3** (**B**), **5** (**C**), and **9** (**D**) for 24 h and compared to control cells (**A**). Cell cycle distribution was determined using DNA cytometry analysis and different cell phases were plotted (**E**) as percentage of total events. Sub-G cell population was taken as representative of total cell death and was plotted as percent of total events (**F**). Data are presented as mean ± SD; *n* = 3. *: significantly different from control group.

Cell cycle distribution using DNA flow cytometry was used to investigate the influence of compounds **3**, **5** and **9** on the proliferation profile of tumor cells. Cells were exposed to compounds **3**, **5** and **9** (1 µM) for 24 h and cell cycle phases were assessed as mentioned in the experimental section. All tested compounds (**3**, **5** and **9**) exerted significant anti-proliferative effect against HCT-116 cells appeared as increased cell population in G_0_/G_1_-phase from 53% ± 1.0% to 59.8% ± 0.2%, 68.4% ± 1.7% and 71.5% ± 0.5% respectively. The increased non proliferating cell population in compounds **3**, **5** and **9** was accompanied by reciprocal decrease in cells in S-phase from 35.4% ± 1.7% to 31.8% ± 0.2%, 21.8% ± 1.2% and 18.7% ± 1.1% respectively. Additionally, only compound **3** treatment resulted in decreasing G_2_/M cells from 11.6% ± 0.9% to 8.4% ± 0.1% after 24 h (Figure 7A–E). In addition, compounds **5** and **9** significantly increased cells in Pre-G phase (apoptotic cells) from 1.0% ± 0.1% to 65.1% ± 1.0% and 4.8% ± 0.1% respectively (Figure 7F). It can be concluded that the potential anticancer activity of compound **3** might be solely attributed to antiproliferative effect while compounds **5** and **9** possess mixed cytotoxic and antiproliferative activities. Further molecular studies on compounds **5** and **9** to assess their modes of action are highly recommended. Due to the low yield of compounds from marine origin on the top of their non-renewability [40], it might be difficult to harvest sufficient material for use in a clinical setting. However, synthetic chemists are highly urged to use this nucleus as a lead compound in the field of anti-cancer drug discovery [41,42,43].

Figure 7 presents the effects of potentially active compounds on cell cycle distribution of HCT-116 cells.

## 3. Experimental Section

### 3.1. General Experimental Procedures

Optical rotation was measured on the automatic high-speed laboratory polarimeter P3000 (A.KRUSS Optronic Gmbh, Hamburg, Germany). UV spectra were measured on a Hitachi 300 Spectrophotometer (Hitachi High-Technologies Corporation, Kyoto, Japan). High-resolution ESIMS data were recorded with an Ultra-High Resolution (UHR) TOF spectrometer (Impact, Bruker, Bremen, Germany). NMR spectra were obtained in CDCl_3_ on a Bruker Avance DRX 600-MHz spectrometer (Bruker) at 600-MHz for ^1^H NMR and 150 MHz for ^13^C NMR. NMR chemical shifts were expressed in parts per million (ppm) referenced to residual CDCl_3_ solvent signals (δ_H_ 7.26 for ^1^H and δ_C_ 77.0 for ^13^C). Precoated SiO_2_ 60 F_254_ plates (Merck, Darmstadt, Germany) were used for TLC. For column chromatography, SiO_2_ (70–230 mesh, Merck) was used. HPLC purifications were performed on HPLC column (5 µm ZORBAX Eclipse XDB-C18, 250 × 4.6 mm, Agilent, Santa Clara, CA, USA).

### 3.2. Biological Materials

Specimen of the marine sponge, *Hyrtios erectus* (Keller, 1889) (Figure 1) was collected from Sharm el-Sheikh, Red Sea Egypt, using scuba diving at a depth of 11 m and 17 m. The sponge material was immediately frozen after collection and kept at −20 °C until investigation. The sponge was kindly identified by Dr. R. van Soest (Institute of Systematic Population Biology, Amsterdam University, The Netherlands) as *Hyrtios erectus* (class: Demospongiae, order: Dictyoceratida, family: Thorectidae). A voucher specimen is kept in the collections of the Zoological Museum of the University of Amsterdam, under ZMAPOR19761 registration number.

### 3.3. Purification of Compounds **1**–**9**

The sponge materials (0.91 kg, wet wt.) were cut into small pieces and were extracted three times at room temperature with MeOH (3 × 2 L). The combined extracts were concentrated under reduced pressure to afford the organic crude extract (86 g). The concentrated total extract was subjected to silica gel column using VLC (vacuum liquid chromatography) stepwise gradient elution (*n*-hexane-CHCl_3_-MeOH) to obtain fractions 1–9. Fraction 4 (*n*-hexane-CHCl3, 1:3) was subjected to silica gel column using *n*-hexane-CHCl_3_-MeOH gradient elution to obtain 8 subfractions (fractions 4-1 to 4-8).

Fraction 4-6 (55 mg) was further subjected to silica gel column chromatography (CC) eluted with *n*-hexane/CHCl_3_ gradient to give 5 subfractions (Fraction 4-6-1 to 4-6-5). Fraction 4-6-3 (14.7 mg) was purified on HPLC (column, XDB-C18 Zorbax, 5 µm, 250 × 4.6 mm, Agilent) using 90% CH_3_CN/H_2_O at a flow rate of 1.5 mL/min and UV detection at 220 nm to yield compound **1** (3.5 mg).

Fraction 4-5 (135 mg) was further subjected to silica gel column chromatography (CC) eluted with *n*-hexane/CHCl_3_ gradient to give 9 subfractions (Fraction 4-5-1 to 4-5-9). Fraction 4-5-6 (34.5 mg) was purified on HPLC (column, XDB-C18 Zorbax, 5 µm, 250 × 4.6 mm) using 90% CH_3_CN/H_2_O at a flow rate of 1.5 mL/min and UV detection at 220 nm to yield compounds **2** (2.3 mg) and **3** (4 mg).

Fraction 4-3 (700 mg) was further subjected to silica gel column chromatography (CC) eluted with *n*-hexane/CHCl_3_ gradient to obtain 10 subfractions (Fraction 4-3-1 to 4-3-10). Fraction 4-3-3 (53 mg) was purified on HPLC (column, XDB-C18 Zorbax, 5 µm, 250 × 4.6 mm) using 80% CH_3_CN/H_2_O at a flow rate of 1.5 mL/min and UV detection at 220 nm to yield compounds **4** (4.6 mg), **5** (14 mg), **6** (3.4 mg), **7** (3 mg) and **8** (4 mg). While, Fraction 4-3-5 (31 mg) was purified on HPLC (column, XDB-C18 Zorbax, 5 µm, 250 × 4.6 mm) using 80% CH_3_CN/H_2_O at a flow rate of 1.5 mL/min and UV detection at 220 nm to yield compound **9** (2.3 mg).

**Compound (1)**: Amorphous solid (3.5 mg); α${}_{D}^{25}$ +81.7 (c 0.20, CHCl_3_); UV (λ_max_, MeOH) (log ε): 226 (4.31), 285 (2.54) nm; NMR data: see Table 1; ESI-MS: *m/z* 461.2 [M + H]⁺. HRESIMS: *m/z* 461.2901 (calculated for C_27_H_41_O_6_ [M + H]⁺, 461.2903).

**Compound (2)**: Amorphous solid (2.3 mg); α${}_{D}^{25}$ −9.7 (c 0.35, CHCl_3_); UV (λ_max_, MeOH) (log ε): 205 (3.74), 252 (3.23) nm; NMR data: see Table 2; ESI-MS: *m/z* 475.3 [M + H]⁺. HRESIMS: *m/z* 475.3057 (calculated for C_28_H_43_O_6_ [M + H]⁺, 475.3060).

### 3.4. Biological Activity of Compounds **1**–**9**

#### 3.4.1. Cell Culture

Human hepatocellular carcinoma cells (HepG2), human breast adenocarcinoma cells (MCF-7), and colorectal adenocarcinoma cells (HCT-116), were obtained from the VACSERA (Giza, Egypt). HepG2 and MCF-7 cells were maintained in DMEM media; and HCT-116 cells were maintained in RPMI-1640 media. All media were supplemented with 100 µg/mL streptomycin, 100 units/mL penicillin and 10% heat-inactivated fetal bovine serum. Cells were propagated in a humidified incubator at 37 °C with 5% (*v/v*) CO_2_ atmosphere.

#### 3.4.2. Trypan-Blue Exclusion Assay

Viability of cells was confirmed prior to seeding using trypan-blue exclusion assay. Briefly, exponentially growing cells were detached using trypsin/EDTA solution. Aliquots of live cell suspensions were exposed to trypan blue solution (0.4%) and percent of trypan blue positive cells was determined. Cell suspensions were not used with viability less than 95%.

#### 3.4.3. Antiproliferative Assessment

The antiproliferative activities of the compounds **1**–**9** on breast adenocarcinoma (MCF-7), colorectal carcinoma (HCT-116) and hepatocellular carcinoma cells (HepG2) were evaluated using the sulforhodamine B (SRB) assay as previously described [44]. Briefly, exponentially growing cells were collected using 0.25% Trypsin-EDTA and plated in 96-well plates at 1000–2000 cells/well. Cells were exposed to serial concentrations of test compounds for 72 h and subsequently fixed with TCA (10%) for 1 h at 4 °C. After washing trice, cells were exposed to 0.4% SRB solution for 10 min in dark place and subsequently washed with 1% glacial acetic acid. After drying overnight, Tris-HCl was used to dissolve the SRB-stained cells and color intensity was measured at 540 nm.

The dose response curve of compounds was analyzed using *E*_max_ model (Equation (1)).

(1) $$\text{\% cell viability}=\left(100-R\right)\times \left(1-\frac{{\left[D\right]}^{m}}{{K_{d}}^{m}+{\left[D\right]}^{m}}\right)+R$$

where (*R*) is the residual unaffected fraction (the resistance fraction), (*D*) is the drug concentration used, (*K_d_*) or IC_50_ is the drug concentration that produces a 50% reduction of the maximum inhibition rate and (m) is a Hill-type coefficient. IC_50_ was defined as the drug concentration required to reduce absorbance to 50% of that of the control (i.e., *K_d_* = absolute IC_50_ when *R* = 0 and *E*_max_ = 100 − *R*) [45].

#### 3.4.4. Apoptosis Assessment Using Annexin V-FITC Staining Coupled with Flowcytometry

To assess the potential of selected active compounds in inducing programmed cell death, apoptosis and necrosis cell populations were determined using Annexin V-FITC apoptosis detection kit (Abcam Inc., Cambridge Science Park, Cambridge, UK). Briefly, HCT-116 cells were treated with compounds **3**, **5** and **9** for 24 h and collected by trypsinization, washed twice with ice-cold PBS, and re-suspended in 0.5 mL of annexin V-FITC/PI solution for 30 min in dark according to manufacturer protocol. After staining at room temperature, cells were injected through ACEA Novocyte™ flow cytometer (ACEA Biosciences Inc., San Diego, CA, USA) and analyzed for FITC and PI fluorescent signals using FL1 and FL2 signal detector, respectively (λ_ex/em_ 488/530 nm for FITC and λ_ex/em_ 535/617 nm for PI). For each sample, 12,000 events were acquired and positive FITC and/or PI cells were quantified by quadrant analysis and calculated using ACEA NovoExpress™ software version 1.1.0 (ACEA Biosciences Inc.).

#### 3.4.5. Analysis of Cell Cycle Distribution

To assess the effect of selected active compounds on cell cycle distribution, HCT-116 cells were treated with compounds **3**, **5** and **9** for 24 h. After treatment, cells were collected by trypsinization; washed twice with ice-cold PBS and re-suspended in 0.5 mL of PBS. Two milliliters of 70% ice-cold ethanol was added gently while vortexing. Cells were kept in ethanol solution at 4 °C for 1 h for fixation. Upon analysis, fixed cells were washed and re-suspended in 1 mL of PBS containing 50 μg/mL RNAase A and 10 μg/mL propidium iodide (PI). After 20 min incubation in dark place at room temperature, cells were analyzed for DNA contents by FACS-VantageTM (Becton Dickinson Immunocytometry Systems). For each sample, 10,000 events were acquired. Cell cycle distribution was calculated using ACEA NovoExpress™ software.

#### 3.4.6. Statistical Analysis

Data are presented as mean ± SEM using GraphPad prism™ software version 5.00 (GraphPad software Inc., La Jolla, CA, USA) for windows version 5.00. Analysis of variance (ANOVA) with Bonferroni post hoc test was used for testing the significance using SPSS^®^ for windows, version 17.0.0. *p* < 0.05 was taken as a cut off value for significance.

## 4. Conclusions

Chemical investigation of the antiproliferative extract of the sponge *Hyrtios erectus*, collected from the Red Sea, Egypt, yielded nine sesterterpenes possessing a scalarane-type framework including two new analogs (**1**) and (**2**) together with the previously reported known compounds 12β,20-dihydroxy-16β-acetoxy-17-scalaren-19,20-olide (**3**) [36], Sesterstatin 7 (**4**) [12], Heteronemin (**5**) [37], Scalarolide (**6**) [17], 12-*epi*-24-deoxyscalarin (**7**) [38], Scalarolide acetate (**8**) [14] and 12-deacetyl-12,18-di-*epi*-scalaradial (**9**) [17]. The structures of isolated compounds were determined by 1D and 2D NMR studies as well as mass spectral determinations. The compounds displayed variable antiproliferative activity against breast adenocarcinoma (MCF-7), colorectal carcinoma (HCT-116), and hepatocellular carcinoma cells (HepG2) using SRB assay. Compounds **3**, **5** and **9** were selected for subsequent investigations regarding their mechanism of cell death induction (differential apoptosis/necrosis assessment) and their influence on cell cycle distribution. It can be concluded that the potential anticancer activity of compound **3** might be solely attributed to antiproliferative effect while compounds **5** and **9** possess mixed cytotoxic and antiproliferative activities.

## Figures and Tables

**Table 1:** NMR data and HMBC (heteronuclear multiple-bond correlation spectroscopy) correlations of compound **1** (CDCl_3_).

Position	δ_C_	δ_H_ (m, *J* in Hz)	HMBC (H→C) ^a^
1	39.7, CH_2_	1.66, 0.79 (m)	C-10
2	18.5, CH_2_	1.60, 1.44 (m)	C-4, C-10
3	41.9 CH_2_	1.37, 1.11 (m)	C-4
4	33.3 qC	-	-
5	56.5 CH	0.78 (m)	C-4
6	18.0 CH_2_	1.59, 1.39 (m)	
7	41.4 CH_2_	1.78, 0.92 (m)	C-8
8	37.3 qC	-	-
9	58.3 CH	0.89 (m)	C-10, C-12
10	37.4 qC	-	-
11	25.8 CH_2_	1.82, 1.55 (m)	C-10, C-12
12	73.8 CH	3.82 (dd, 16.8, 6.6)	C-9, C-11, C-13, C-25, C-26
13	44.6 qC	-	-
14	53.3 CH	1.25 (m)	C-8, C-9, C-13, C-16, C-18
15	24.2 CH_2_	2.20, 1.63 (m)	C-8, C-13, C-14, C-16, C-17
16	65.9 CH	5.67 (dd, 9.6, 7.2)	C-15, C-17, C-20
17	126.1 qC	-	-
18	168.7 qC	-	-
19	95.5 CH	6.17 (s)	C-17, C-18
20	170.8 qC	-	-
21	21.2 CH_3_	0.80 (s)	C-4
22	33.2 CH_3_	0.84 (s)	C-4
23	17.5 CH_3_	0.89 (s)	C-7, C-8, C-9, C-14
24	16.1 CH_3_	0.84 (s)	C-1, C-5, C-9, C-10
25	16.7 CH_3_	1.23 (s)	C-12, C-13, C-14, C-18
26	169.8 qC	-	-
27	21.0 CH_3_	2.09 (s)	C-26

^a^: HMBC correlations are from proton(s) stated to the indicated carbons.

**Table 2:** NMR data and HMBC correlations of compound **2** (CDCl_3_).

Position	δ_C_	δ_H_ (m, *J* in Hz)	HMBC (H→C) ^a^
1	39.5, CH_2_	1.61, 0.82 (m)	C-10
2	18.4, CH_2_	1.60, 1.42 (m)	C-4, C-10
3	42.0 CH_2_	1.36, 1.11 (m)	C-4
4	33.2 qC	-	-
5	56.5 CH	0.79 (m)	C-4
6	18.1 CH_2_	1.58, 1.42 (m)	-
7	41.6 CH_2_	1.84, 0.93 (m)	C-8
8	37.1 qC	-	-
9	57.7 CH	0.96 (m)	C-10, C-12
10	37.3 qC	-	-
11	24.4 CH_2_	1.74, 1.55 (m)	C-10, C-12
12	75.9 CH	4.88 (dd, 10.8, 3.6)	C-13, C-18, C-25, C-26
OH	-	4.25 (br s)	-
13	41.5 qC	-	-
14	54.2 CH	1.15 (m)	C-25
15	23.2 CH_2_	2.29, 1.48 (m)	-
16	74.5 CH	4.08 (dd, 9.0, 6.6)	C-17, C-18, C-28
17	159.4 qC	-	-
18	138.3 qC	-	-
19	167.8 qC	-	-
20	94.2 CH	5.98 (s)	C-17, C-18, C-19
21	21.2 CH_3_	0.80 (s)	C-3, C-4, C-5
22	33.3 CH_3_	0.84 (s)	C-3, C-4, C-5, C-21
23	17.4 CH_3_	0.92 (s)	C-7, C-8, C-9, C-14
24	15.9 CH_3_	0.82 (s)	C-1, C-5, C-9, C-10
25	16.6 CH_3_	1.26 (s)	C-12, C-13, C-14, C-18
26	171.9 qC	-	-
27	21.8 CH_3_	2.13 (s)	C-27
28	57.5 OCH_3_	3.47 (s)	C-16

^a^: HMBC correlations are from proton(s) stated to the indicated carbons.

**Table 3:** Antiproliferative activity of compounds **1**–**9** in vitro (IC_50_, µM) against a series of human tumor cell lines.

Cell Type	Cell Line	1	2	3	4	5	6	7	8	9
Breast	MCF-7	32.6	40.3	12.7	24.0	1.1	>100	30.7	20.9	3.3
Colorectal	HCT-116	57.5	22.5	3.5	26.1	0.7	>100	14.4	15.4	3.4
Hepatocellular	HepG2	21.8	42.5	9.6	19.2	1.1	>100	17.3	15.5	1.7

Doxorubicin positive cytotoxic control.

## Supplementary Materials

The following are available online at www.mdpi.com/1660-3397/14/7/130/s1, Figures S1: ^1^H-NMR spectrum of compound **1** (CDCl_3_), Figures S2: ^13^C-NMR spectrum of compound **1** (CDCl_3_), Figures S3: HSQC spectrum of compound **1** (CDCl_3_), Figures S4: HMBC spectrum of compound **1** (CDCl_3_), Figures S5: ^1^H-^1^H COSY spectrum of compound **1** (CDCl_3_), Figures S6: NOESY spectrum of compound **1** (CDCl_3_), Figures S7: ^1^H-NMR spectrum of compound **2** (CDCl_3_), Figures S8: ^13^C-NMR spectrum of compound **2** (CDCl_3_), Figures S9: HSQC spectrum of compound **2** (CDCl_3_), Figures S10: HMBC spectrum of compound **2** (CDCl_3_), Figures S11: ^1^H-^1^H COSY spectrum of compound **2** (CDCl_3_), Figures S12: NOESY spectrum of compound **2** (CDCl_3_), Table S1: Influence of compounds **3**, **5** and **9** on the membrane integrity of HCT116 cells after exposure to their pre-determined IC_50_’s for 72 h.
