# Supplementary material for: Antiproliferative Scalarane-Based Metabolites from the Red Sea Sponge Hyrtios erectus
Source: Mar Drugs. 2016 Jul 8;14(7):130. doi: 10.3390/md14070130 (PMC4962020; doi:10.3390/md14070130)
Supplement: Supplementary file 1 [file marinedrugs-14-00130-s001.pdf]

Supplementary Materials: Antiproliferative Scalarane-Based Metabolites from the Red Sea Sponge *Hyrtios erectus*

Sameh S. Elhady, Ali M. El-Halawany, Ahmed M. Al-Abd, Abdulrahman M. Alahdal, Hashim A. Hassanean and Safwat A. Ahmed

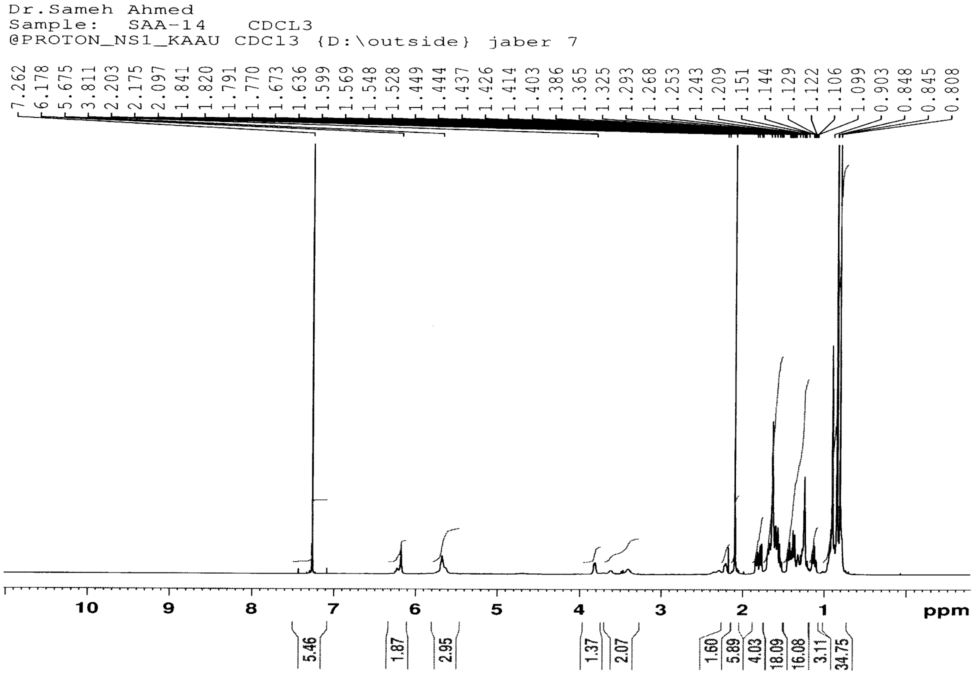

Figure S1. ^{1}H-NMR spectrum of compound **1** ($CDCl_3$).

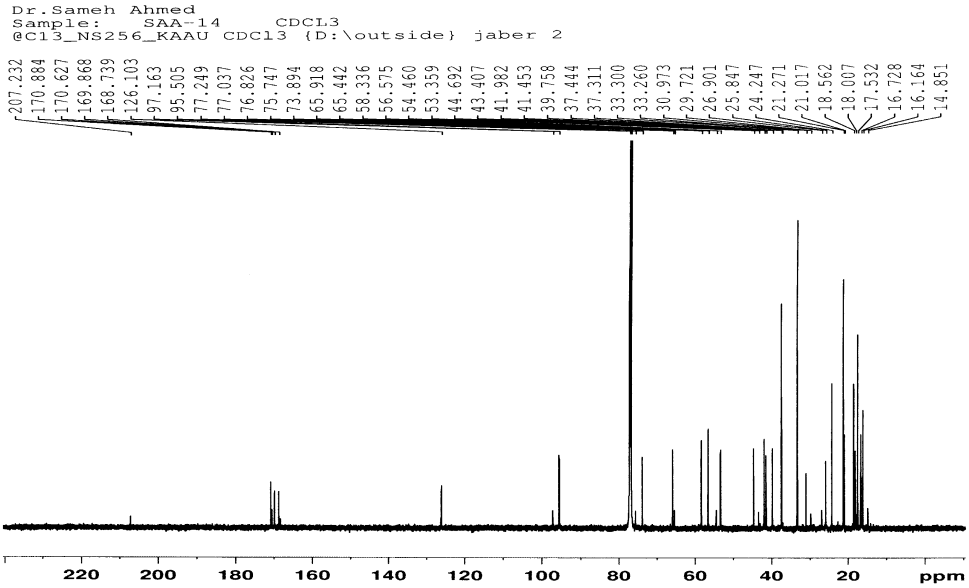

Figure S2. ^{13}C-NMR spectrum of compound **1** ($CDCl_3$).

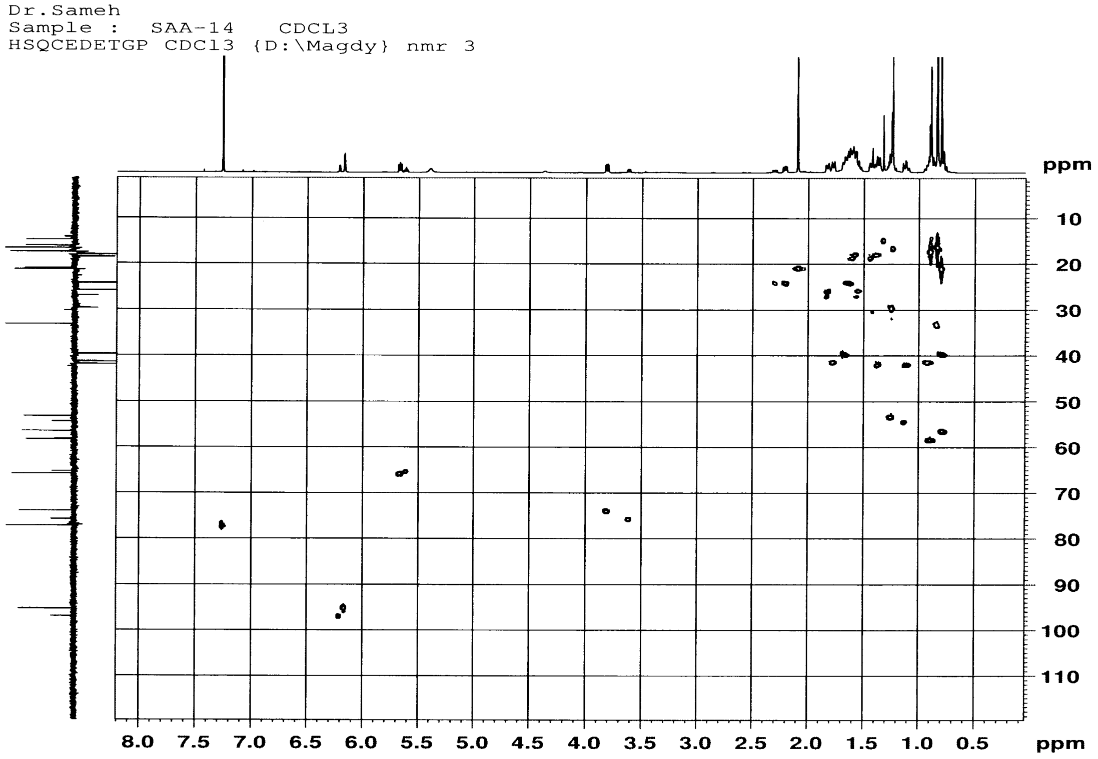

Figure S3. HSQC spectrum of compound **1** ($CDCl_3$).

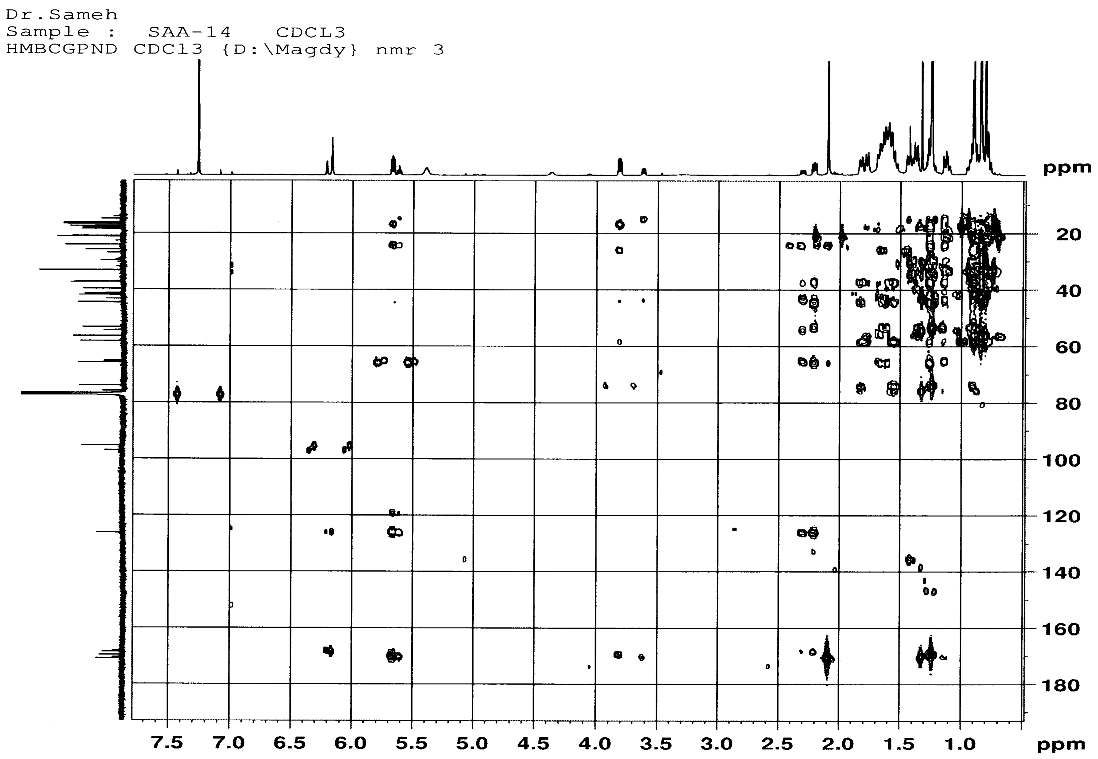

Figure S4. HMBC spectrum of compound **1** ($CDCl_3$).

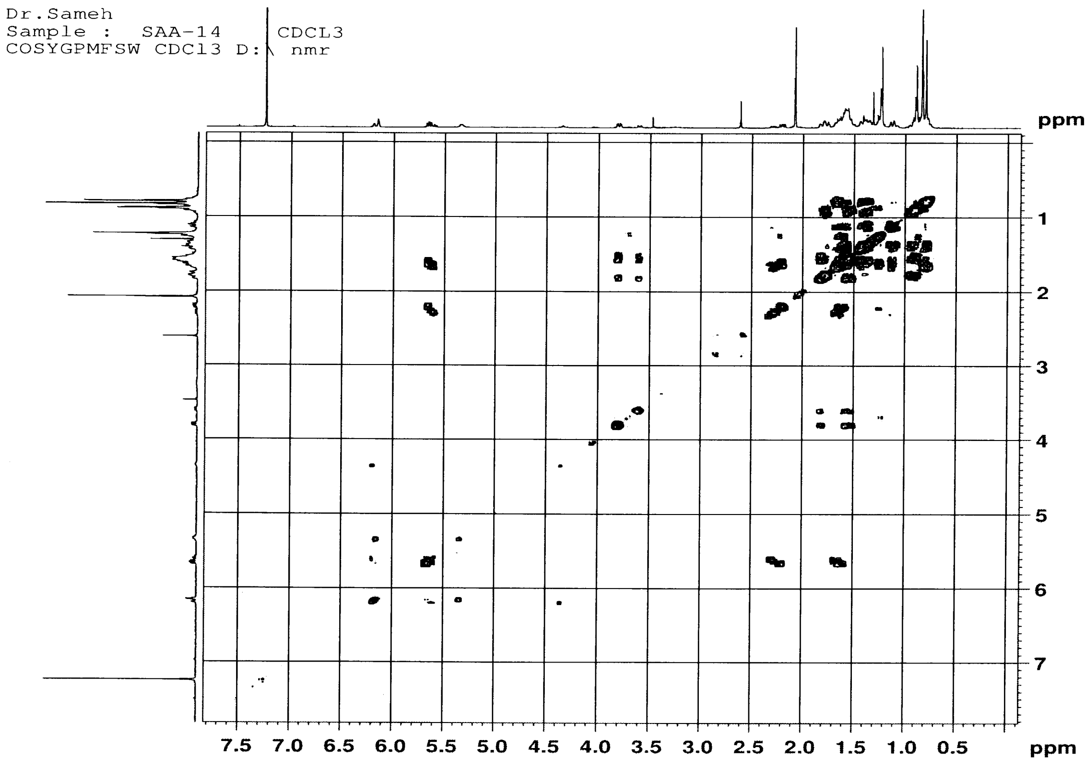

Figure S5. ^1H-^1H COSY spectrum of compound **1** ($CDCl_3$).

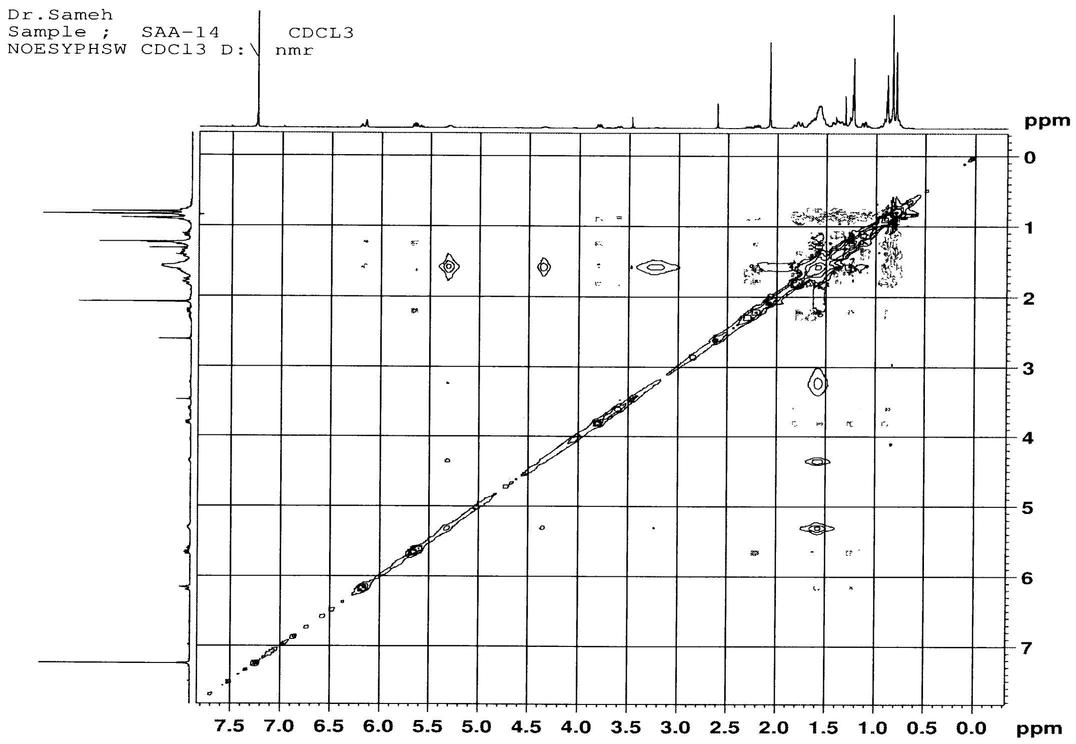

Figure S6. NOESY spectrum of compound **1** ($CDCl_3$).

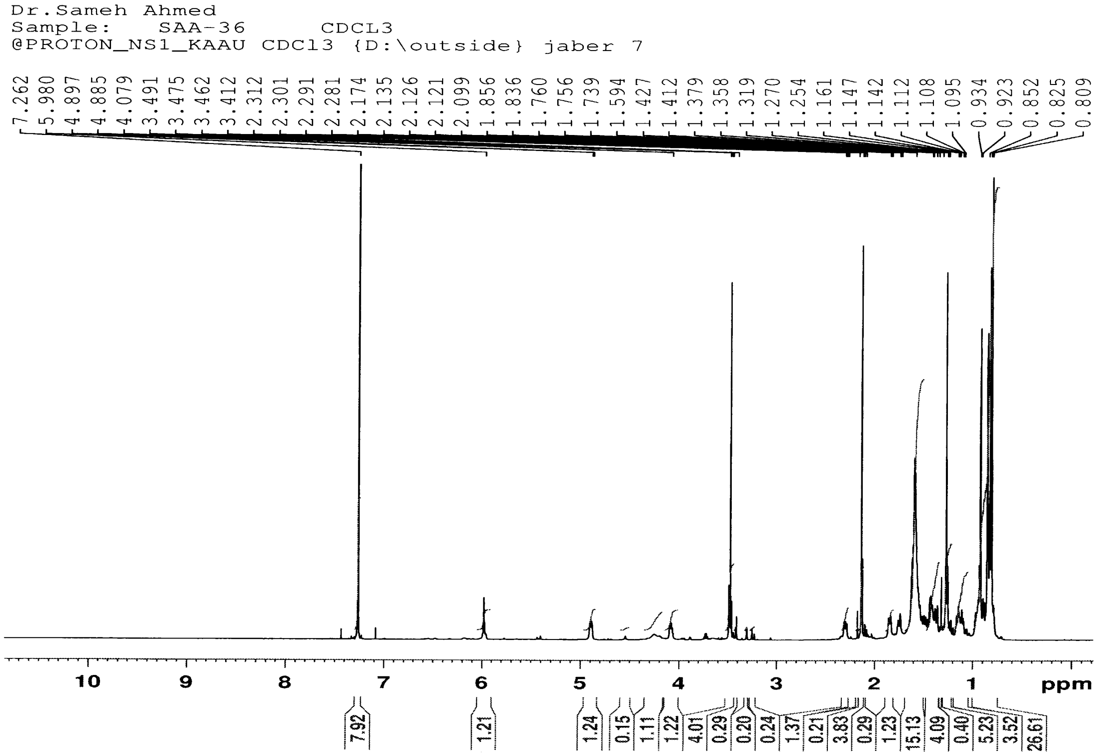

Figure S7. ^{1}H-NMR spectrum of compound **2** ($CDCl_3$).

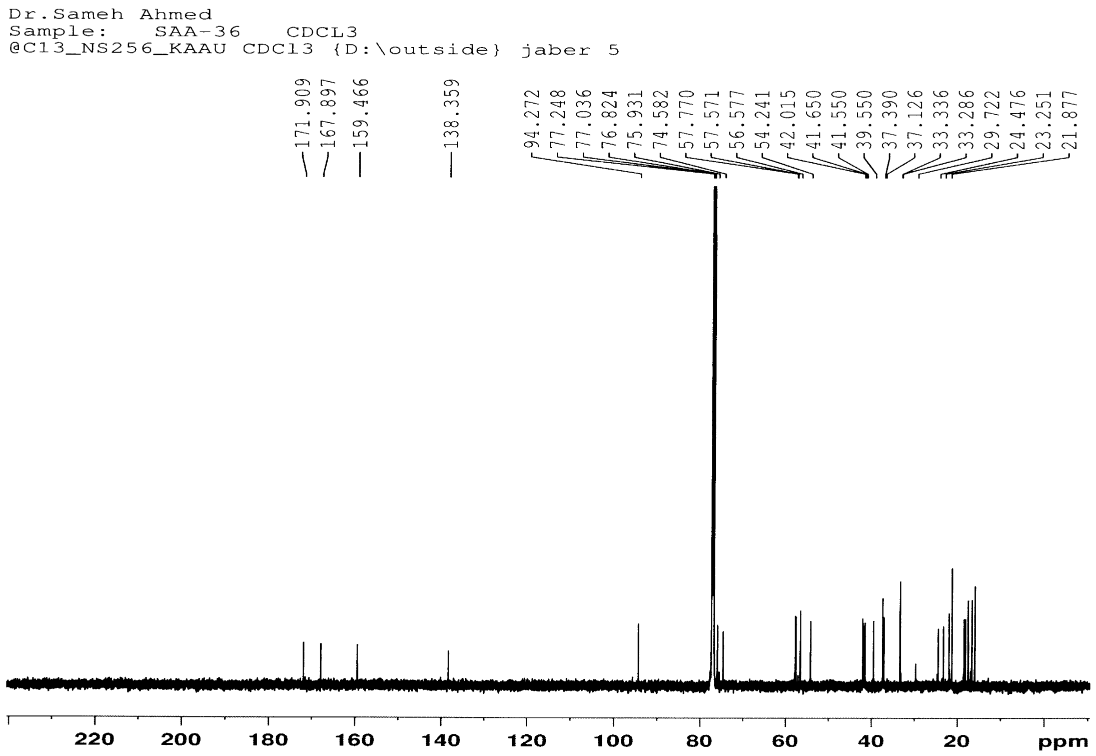

Figure S8. ^{13}C-NMR spectrum of compound **2** ($CDCl_3$).

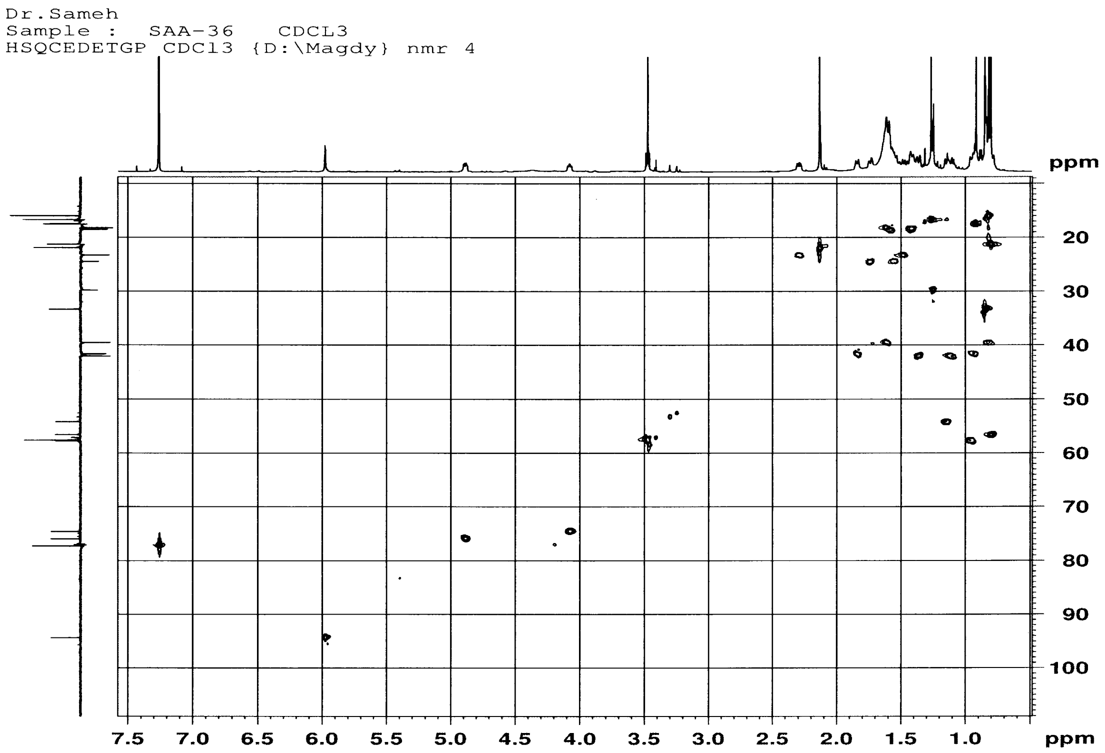

Figure S9. HSQC spectrum of compound **2** ($CDCl_3$).

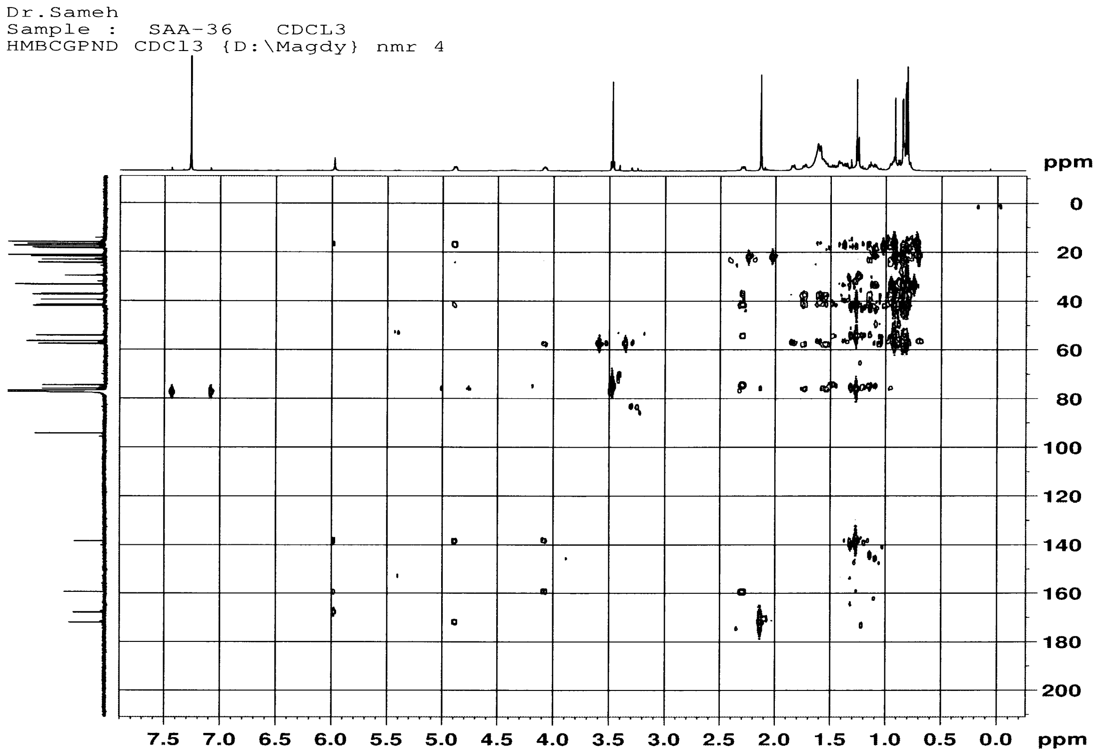

Figure S10. HMBC spectrum of compound **2** ($CDCl_3$).

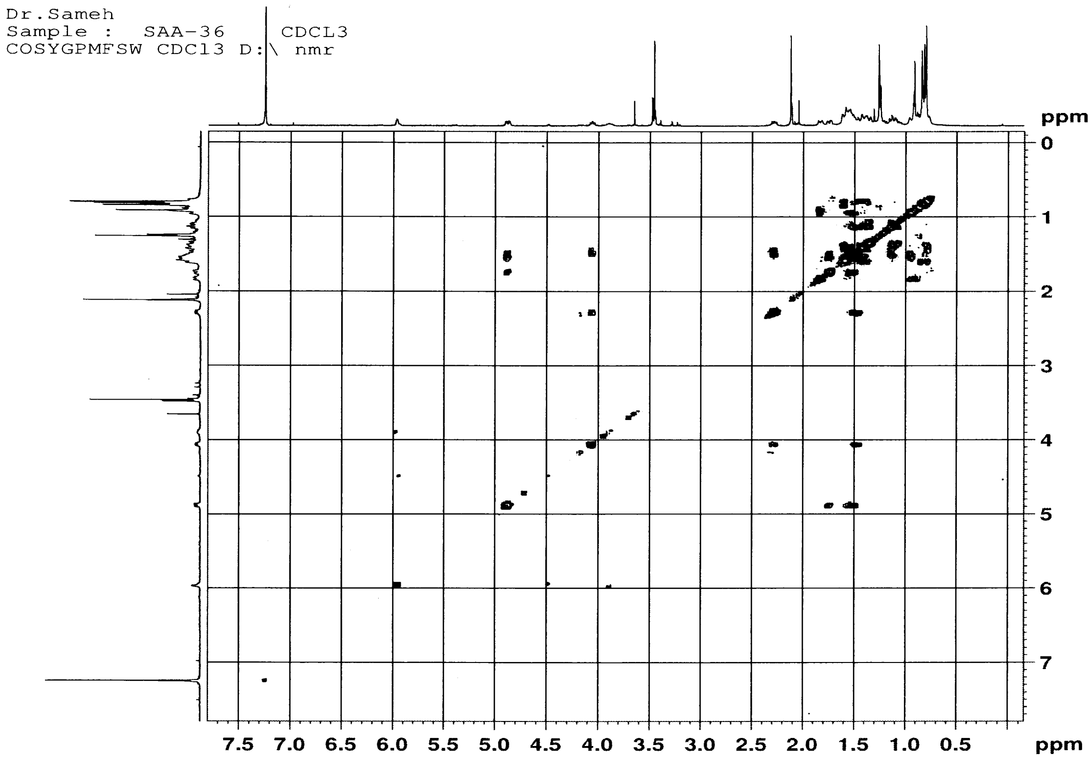

Figure S11. ^1H-^1H COSY spectrum of compound **2** ($CDCl_3$).

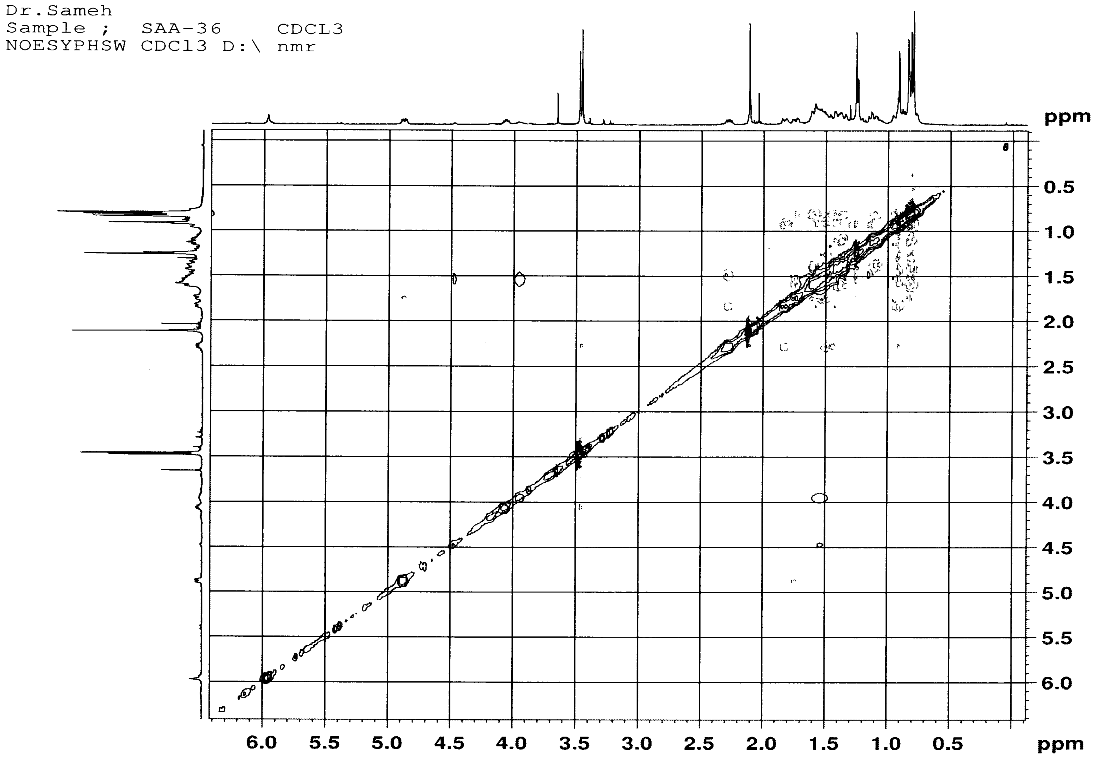

Figure S12. NOESY spectrum of compound **2** ($CDCl_3$).

Table S1. Influence of compounds **3**, **5** and **9** on the membrane integrity of HCT116 cells after exposure to their pre-determined IC_{50}'s for 72 h.

Treatment	Trypan Blue Positive Cells (%)
Compound **3**	5.1% ± 1.3%
Compound **5**	67.6% ± 4.2%
Compound **9**	9.5% ± 2.1%
